# Plasma HIV-2 RNA According to CD4 Count Strata among HIV-2-Infected Adults in the IeDEA West Africa Collaboration

**DOI:** 10.1371/journal.pone.0129886

**Published:** 2015-06-25

**Authors:** Didier K. Ekouévi, Véronique Avettand-Fènoël, Boris K. Tchounga, Patrick A. Coffie, Adrien Sawadogo, Daouda Minta, Albert Minga, Serge P. Eholie, Jean-Christophe Plantier, Florence Damond, François Dabis, Christine Rouzioux, IeDEA West Africa collaboration

**Affiliations:** 1 Université de Bordeaux, ISPED, Centre INSERM U897- Epidémiologie-Biostatistique, Bordeaux, France; 2 Inserm U897, ISPED, Université de Bordeaux, Bordeaux, France; 3 Programme PACCI, site de recherche ANRS, Abidjan, Côte d’Ivoire; 4 Département de Santé Publique, Université de Lomé, Lomé, Togo; 5 AP-HP, Hôpital Necker Enfants Malades, Laboratoire de Virologie, Paris, France; 6 Université Paris-Descartes, Sorbonne Paris Cité, Faculté de Médecine, EA7327, Paris, France; 7 Service des Maladies Infectieuses et Tropicales, CHU de Treichville, Abidjan, Côte d’Ivoire; 8 Hôpital de Jour, Service des Maladies Infectieuses et Tropicales, CHU SouroSanou, Bobo Dioulasso, Burkina-Faso; 9 Centre de Prise en Charge des Personnes vivant avec le VIH, Hôpital du Point G, Bamako, Mali (service des Maladies Infectieuses); 10 Centre Médical de Suivi de Donneurs de Sang/CNTS/PRIMO-CI, Abidjan, Côte d’Ivoire; 11 Laboratoire associé au Centre National de Référence du VIH, hôpital Charles Nicolle, CHU de Rouen, Rouen, France; 12 GRAM, Equipe d’Accueil 2656, Faculté de Médecine-Pharmacie, Institut de Recherche et d'Innovation en Biomédecine, Université de Rouen, Rouen, France; 13 INSERM, IAME, UMR 1137, Paris, France; 14 Université Paris Diderot, Sorbonne Paris Cité, Paris, France; 15 AP-HP, Hôpital Bichat-Claude Bernard, Laboratoire de Virologie, Paris, France; CEA, FRANCE

## Abstract

**Background:**

Plasma HIV-1 RNA monitoring is one of the standard tests for the management of HIV-1 infection. While HIV-1 RNA can be quantified using several commercial tests, no test has been commercialized for HIV-2 RNA quantification. We studied the relationship between plasma HIV-2 viral load (VL) and CD4 count in West African patients who were either receiving antiretroviral therapy (ART) or treatment-naïve.

**Method:**

A cross sectional survey was conducted among HIV-2-infected individuals followed in three countries in West Africa from March to December 2012. All HIV-2 infected-patients who attended one of the participating clinics were proposed a plasma HIV-2 viral load measurement. HIV-2 RNA was quantified using the new ultrasensitive in-house real-time PCR assay with a detection threshold of 10 copies/ mL (cps/mL).

**Results:**

A total of 351 HIV-2-infected individuals participated in this study, of whom 131 (37.3%) were treatment naïve and 220 (62.7%) had initiated ART. Among treatment-naïve patients, 60 (46.5%) had undetectable plasma HIV-2 viral load (<10 cps/mL), it was detectable between 10-100 cps/mL in 35.8%, between 100-1000 cps/mL in 11.7% and >1000 cps/mL in 6.0% of the patients. Most of the treatment-naïve patients (70.2%) had CD4-T cell count ≥500 cells/mm^3^ and 43 (46.7%) of these patients had a detectable VL (≥10 cps/mL). Among the 220 patients receiving ART, the median CD4-T cell count rose from 231 to 393 cells/mm^3^ (IQR [259-561]) after a median follow-up duration of 38 months and 145 (66.0%) patients had CD4-T cell count ≤ 500 cells/mm^3^ with a median viral load of 10 cps/mL (IQR [10-33]). Seventy five (34.0%) patients had CD4-T cell count ≥ 500 cells/mm^3^, among them 14 (18.7%) had a VL between 10-100 cps/mL and 2 (2.6%) had VL >100 cps/mL.

**Conclusion:**

This study suggests that the combination of CD4-T cell count and ultrasensitive HIV-2 viral load quantification with a threshold of 10 cps/mL, could improve ART initiation among treatment naïve HIV-2-infected patients and the monitoring of ART response among patients receiving treatment.

## Introduction

Infection due to Human Immune deficiency Virus type 2 (HIV-2) is predominantly found in West Africa and in countries with historical ties to this part of the world [1]. This infection is characterized by its low sexual [2] and vertical transmission rate [3] and its slow clinical and immunological progression [4–6]. However, HIV-2 infection can lead to acquired immune deficiency syndrome (AIDS) [6,7] and its treatment is challenging due to the natural resistance of HIV-2 to non-nucleoside reverse transcriptase inhibitor (NNRTI) [8] and to some protease inhibitors (PI) [9–11]. The successful scaling up of antiretroviral therapy (ART), as a global response to the epidemic [12] has made viral load monitoring a new challenge in the management of HIV-infected people. Many surveys involving ART-naïve and on-ART West African patients have reported primary and post-therapeutic viral resistance to most of the molecules used in the World Health Organization (WHO)-recommended ART regimen for HIV-2 infection [13,14], underlining the importance of an intensive monitoring of ART response among HIV-2-infected individuals.

Plasma HIV-1 RNA quantification is the gold standard test for the determination of success or failure of antiretroviral treatment (ART) [8]. While HIV-1 ribonucleic acid (RNA) can be quantified using several commercial tests, no test has been commercialized for HIV-2 viral load quantification so far [15–18]. With most in-house tests, a high percentage of HIV-2-infected patients have undetectable pre-ART viral load [15]. In Europe, HIV-2 RNA is detectable in about 61% of infected individuals at treatment initiation [19] and another survey in United Kingdom found that only 8% of the patients with CD4 count > 500 cells/mm^3^ had detectable HIV-2 viral load and 38% of patients with CD4 count <300 cells/mm^3^ had detectable HIV-2 viral load [18]. These results are consistent with the fact that HIV-2 has a lower viral replication level than HIV-1, implying that plasma HIV-2-RNA quantification assays should be very sensitive, in order to be used for ART monitoring. Recently, the French National Research Agency on AIDS and viral hepatitis (ANRS) has developed a new ultrasensitive plasma HIV-2 viral load quantification assay [20]. This test is presently being used for HIV-2-infected patients in the French HIV-2 cohort and its implementation for research purpose is ongoing in few referral laboratories in the West African region, known to be the epicentre of HIV-2 epidemic [1].

In 2011, the International epidemiological Database to Evaluate AIDS (IeDEA) West Africa Collaboration (WADA), which is part of the global IeDEA network initiated the HIV-2 cohort of patients (WADA HIV-2) [21,22]. Currently, this cohort includes 4970 HIV-2 and HIV-1&2 dually reactive patients. In order to improve the clinical management including the monitoring of ART response and the early detection of ART failure among patients followed in our cohort, we measured plasma HIV-2 viral load and analyzed the relationship between this plasma HIV-2 viral load and lymphocytes T-CD4 count in West African patients who are either on ART or treatment-naïve, by using the new ultrasensitive in-house test validated in France [20].

## Method

### Study design

A cross-sectional survey was conducted from March to December 2012 in Burkina Faso, Côte d’Ivoire and Mali among HIV-2-infected patients, followed up in the clinical sites participating in the IeDEA West Africa collaboration [22].

### Study sample

All patients who were 18 years and above, registered in the WADA HIV-2 database, and attended one of the participating clinics during the study period were invited to participate in this survey regardless of ART initiation.

### Data collection

A standardized survey form was used to collect data about patient’s demographics and biological characteristics from the enrollment in the cohort until the time of the study.

### Biological tests

In this study, only HIV-2 mono-infected patients were included based on a two-level discrimination process. The first level was at the clinical sites, where HIV diagnosis and HIV type discrimination was made using the national algorithm of each country. These algorithms recommended the of at least two rapid tests performed in series and in case of discordant results, the use of a third test, usually an Elisa or western blot test. All patients identified as HIV-2 on clinical site based on these national algorithms were screened de novo with two immuno-enzymatic tests: ImmunocombII (HIV-1&2 ImmunoComb BiSpot—Alere), a WHO endorsed indirect, immuno-enzymatic test (sensitivity 100%; specificity 99%) [23] and an in-house enzyme-link immunosorbent assay (ELISA) test, developed by the ANRS [24]. Only patients confirmed as HIV-2 infected based on these tests were included in the survey. The results based on the screening of HIV-2-infected patients in the WADA HIV-2 database were previously reported [25,26]. Two EDTA tubes of blood were collected from each patient and sent to the referral laboratory of the study (CeDReS, Treichville Hospital in Abidjan, Côte d’Ivoire). The first blood sample tube from each participant was used to perform the absolute CD4+/CD8+ T-cell counts using flow cytometry standard (FACScan, Becton Dickinson) at the referral laboratory in Abidjan. The second blood sample tube was frozen at -80°C and sent to the Virology Laboratory of Necker’s hospital in Paris (France) where plasma HIV-2 RNA quantification was performed using a new ultrasensitive in-house real-time polymerase chain reaction (PCR) assay, developed and validated by the AC11Quantification Group of the ANRS [20]. This quantification assay was performed using 1 ml of EDTA plasma samples to achieve a threshold of 10 cps/ mL

### Antiretroviral treatment

Antiretroviral treatment was provided to the HIV-2-infected patients according to each national guideline. In West Africa, HIV-2 treatment guidelines were based on WHO recommendations [8]. Before 2010, the first-line regimen was constituted of two NRTIs (zidovudine or stavudine (d4T) plus lamivudine (3TC) or didanosine (DDI) and a PI boosted or unboosted (indinavir, saquinavir or lopinavir). Three NRTIs regimens should be prescribed as an alternative regimen in case of tuberculosis and/or for HIV-2-infected patients with CD4 count >200 cells/mm^3^ [27]. Since 2010, boosting PI was clearly recommended and lopinavir chosen as the preferred option. Three NRTIs regimen was indicated when boosting PI was contraindicated or not tolerated. The suggested combinations are zidovudine plus lamivudine or emtricitabine plus tenofovir or abacavir [8].

### Data analysis

The Chi-square or Fisher exact tests were used to compare proportions while the non-parametric test of Kruskall-Wallis was used for the comparison of median values and distributions of quantitative variables. The Pearson correlation test was used to explore the correlation between HIV-2 viral load quantification and CD4 count strata. Data analysis was performed using STATA software (STATA 9.0 College Station, Texas, USA).

### Ethics

This survey was approved by the national ethics committee of each participating country. The “Comité d’Ethique pour la Recherche en Santé au Burkina Faso” (CERS_BF) approved the study in Burkina-Faso, the “Comité National pour l’Ethique et la Recherche en Sante” (CNER_CI) approved the study in Côte d’Ivoire and the “Comité National d’Ethique pour la Santé et les Sciences de la vie” approved the study in Mali. All patients were informed and had to give their written consent before being included.

## Results

### Demographic characteristics

A total of 351 HIV-2-infected individuals were enrolled in this study. Among them, 131 (37.3%) were treatment-naïve and 220 (62.7%) had initiated ART. The median age was 48 years (inter-quartile range IQR [40–54]) and there were 202 (57.2%) women. The main baseline and follow-up characteristics of the study population are summarized in Table 1.

Among the 131 treatment-naïve patients, the median CD4-T cell count at enrolment in the cohort was 601 cells/mm^3^ and did not vary much over time, moving to 619 cells/mm^3^ at the time of blood collection, after a median follow-up duration of 27 months (Table 1). Among the 220 patients receiving ART, the median CD4-T cell count at treatment initiation was 231 cells/mm^3^ (IQR [131–395]) and 393 cells/mm^3^ at the time of sampling. The first-line ART regimen was mainly based on an association of 2 NRTI and a boosted PI (79.0%). Lopinavir/ritonavir was the most prescribed boosted PI (initiated in 66.1% of patients), followed by Indinavir/ritonavir (33.9%). Fourteen patients (6.4%) received 3 NRTI combinations, mainly AZT+3TC+abacavir (86.0%) and 14.6% of the patients in the cohort initiated NNRTI–based regimen.

**Table 1:** Demographic and follow-up characteristics of HIV-2 infected patients in the IeDEA-WA HIV-2 cohort.

	On ART		ART naïve		Total		P values
	220	(62.7%)	131	(37.3%)	351		
Age at date of sampling							
Median	49		46		48		0.008
IQR	[43–54]		[39–52]		[40–54]		
Min-max	18–76		20–66		18–76		
Gender							**0.032**
Male	103	(46.8)	46	(35.1)	149	(42.5)	
Female	117	(53.2)	85	(64.9)	202	(57.5)	
CD4 count at date of enrolment (cells/μL)							
Median	231		601		353		<0.000
IQR	[131–395]		[461–905]		[181–590]		
Min-max	4–1564		9–2281		4–2281		
CD4 count at date of sampling (cells/μL)							
Median	393		619		487		<0.000
IQR	[256–561]		[419–855]		[319–667]		
Min-max	35–2077		9–1885		4–2077		
% CD4 cells at date of sampling							
Median	21.2		36.3		26.9		<0.000
IQR	[14.9–29.5]		[28.3–41.5]		[17.4–35.0]		
Min-max	2.3–65.4		0.6–67.4		0.6–67.4		
Follow up duration at date of sampling (months)							
Median	38		27		35		0.040
IQR	[9–71]		[8–64]		[8–69]		
Min-max	0–163		0–179		0–179		
Country							
Côte d’Ivoire	150	(68.2)	116	(88.5)	266	(75.8)	
Burkina Faso	51	(23.2)	12	(9.2)	63	(17.9)	
Mali	19	(8.6)	3	(2.3)	22	(6.3)	

ART: antiretroviral therapy; % (percentage), IQR: inter-quartile Range, Min: Minimum, Max: Maximum

### Detection of HIV-2 viral load

With a threshold of 10 cps/mL the viral load was detectable in 146 (41.6%) of the 351 samples tested. When thresholds of 50 and 100 cps/mL were used, the viral load was detectable in only 65 (18.5%) and 59 (16.8%) samples respectively.

### HIV-2 viral load and link with CD4 count

#### Treatment naïve patients

**Fig 1:**
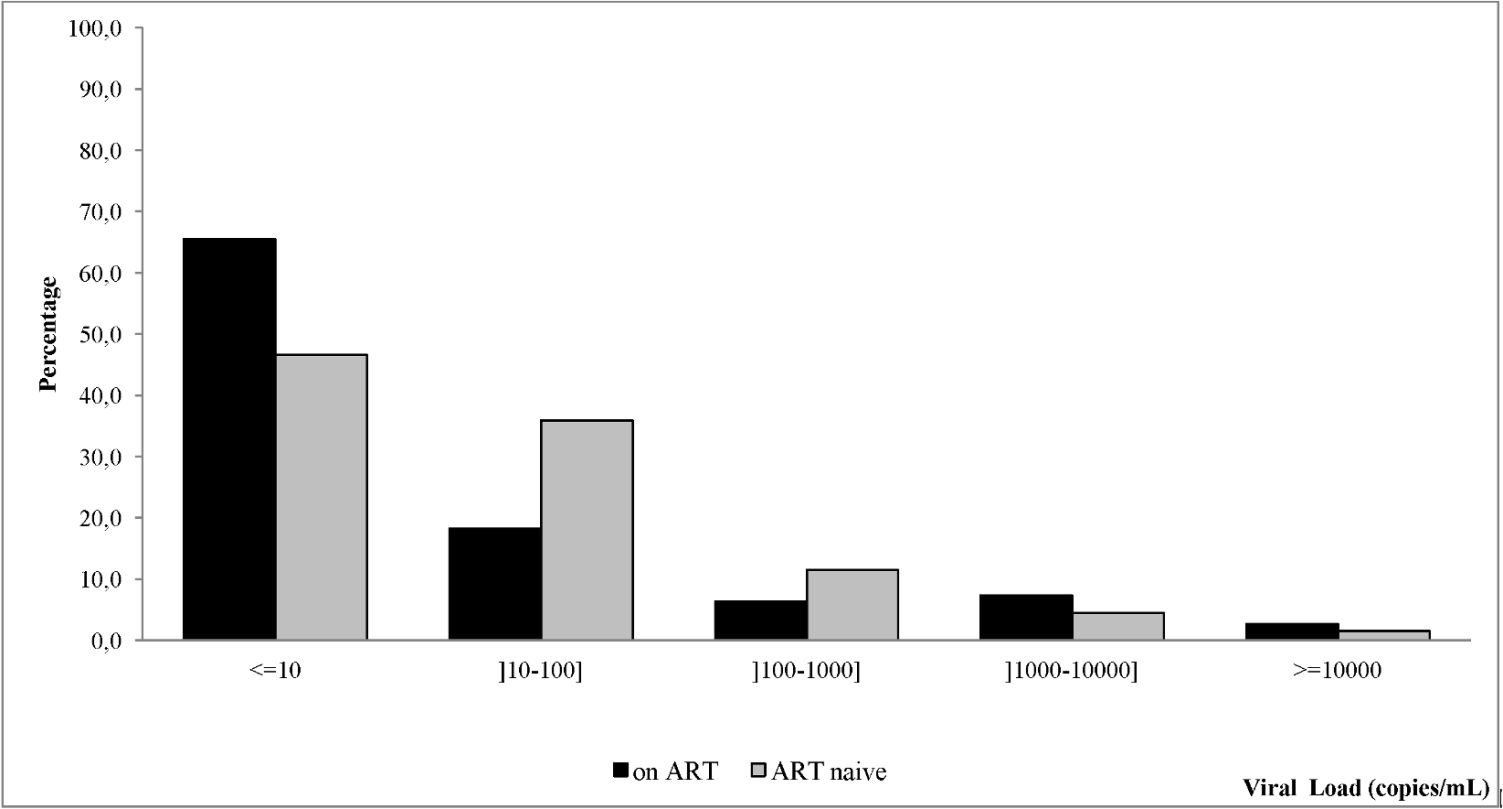
Distribution of viral load according to antiretroviral therapy initiation among HIV-2 infected individuals in West Africa.

**Fig 2:**
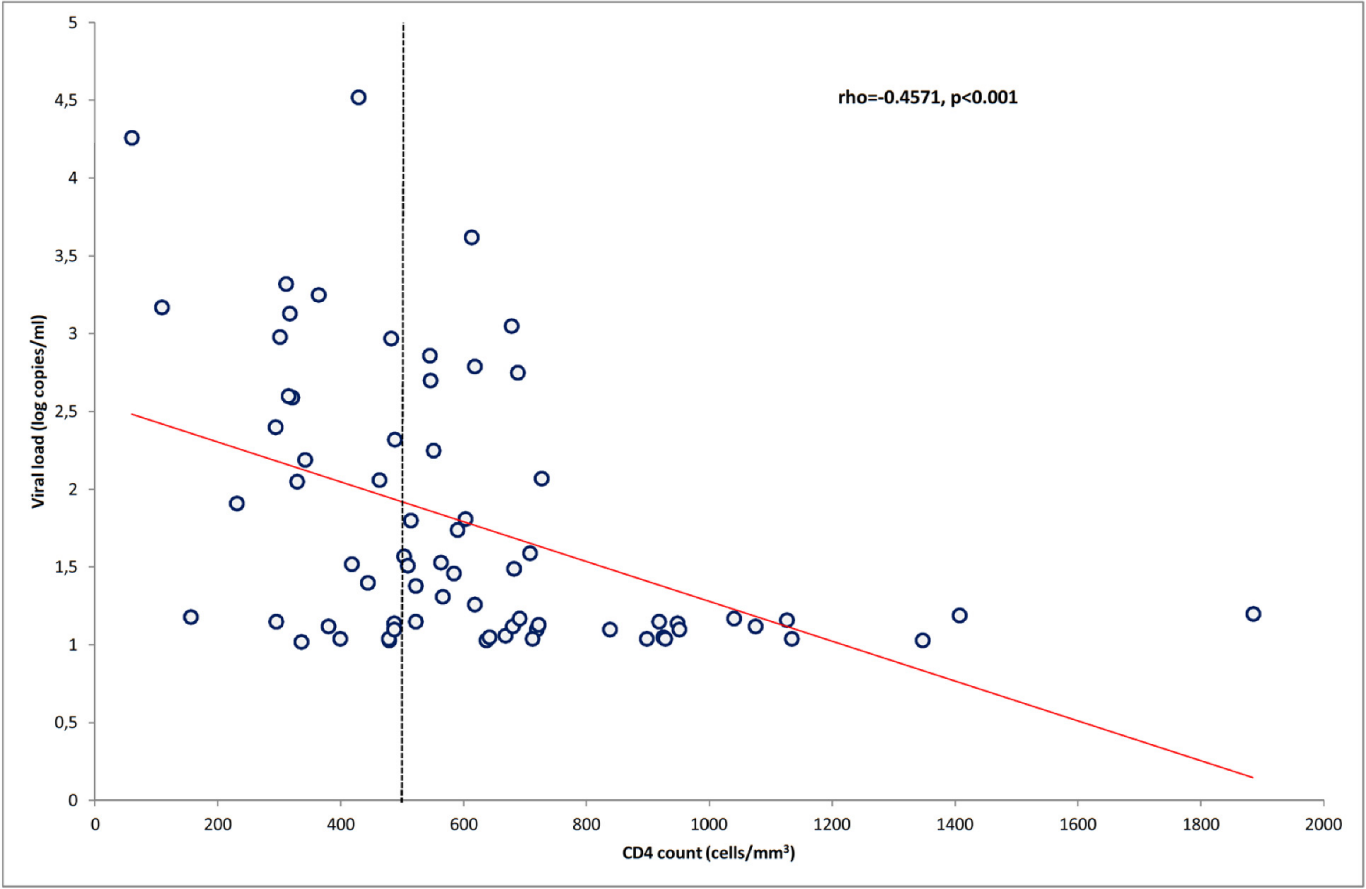
Correlation between plasma HIV-2 RNA and CD4 count among naive HIV-2 infected individuals in West Africa.

Sixty (46.5%) treatment-naïve patients had undetectable plasma HIV-2 viral load (<10 cps/mL), 35.8% had it between 10–100 cps/mL, 11.7% between 100–1000 cps/mL and 6.0% >1000 cps/mL (Fig 1). The median viral load in patients with detectable HIV-2 viral load was 31.8 cps/mL, IQR [12.5–712.8]. Among the 131 ART-naïve patients, 92 (70.2%) had a CD4-T cell count ≥ 500 cells/mm^3^ and within them 43 (46.7%) had a detectable viral load (≥10 cps/mL) including 35 (81.5%) between 10 and 100 cps/mL. For the 39 (29.8%) treatment-naïve patients with CD4-T cell count <500 cells/mm^3^, the viral load was detectable in 70.0% of them. The proportion of patients with detectable viral load decreases when CD4-T cell count increases. Fig 2 shows an inverse correlation between plasma HIV-2 viral load and CD4-T cell count in treatment-naïve patients (ρ = -0.457; p<0.001).

#### Patients receiving ART

Among the 220 patients receiving ART, the median CD4 count rose from 231 cells/mm^3^ at ART initiation to 393 cells/mm^3^ (IQR [259–561]) after a median duration on ART of 38 months (IQR [40–54]) and 145 (66.0%) patients had a CD4 count < 500 cells/mm^3^. The viral load was undetectable (<10 cps/mL) for 144 (65.5%) patients, 40 (18.8%) other patients had a viral load between 10–100 cps/mL, 14 (7.3%) between 100–1000 cps/mL and 22 (10.0%) >1000 cps/mL (Fig 1). Among the 220 patient receiving ART, 210 received it more than six months and 72 (34.3%) of them had viral load ≥10 cps/mL. Ten patients out of 220 (4.5%) received ART less than 6 months and 4 (40%) of them had a detectable viral load (>10 copies).

**Fig 3:**
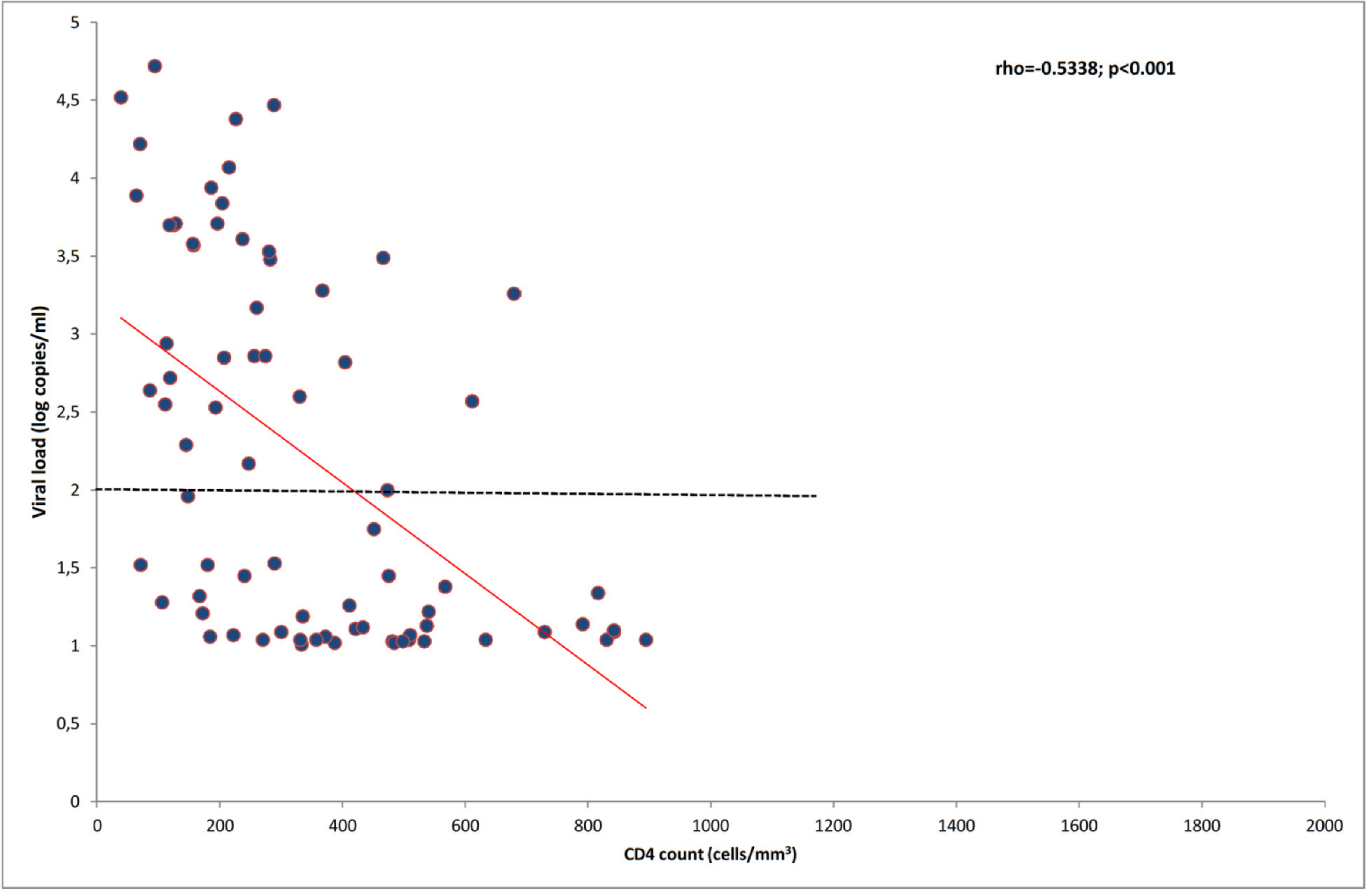
Correlation between plasma HIV-2 RNA and CD4 count among HIV-2 infected individuals on antiretroviral therapy in West Africa.

Among the 75 (34.0%) patients with CD4-T cell count ≥ 500 cells/mm^3^, 14 (18.7%) had a viral load between 10–100 cps/mL and 2 (2.6%) had viral load > 100 cps/mL. Fig 3 shows an inverse correlation between plasma HIV-2 viral load and CD4-T cell count in patients receiving ART (ρ = -0.533; p<0.001).

## Discussion

This study quantified plasma HIV-2 viral load in 351 plasma samples from West African HIV-2-infected individuals using an ultra-sensitive procedure (threshold 10 cps/mL) of the new HIV-2 quantification assay developed by the ANRS [26]. We first described HIV-2 viral load in treatment naive patients and showed a low median level of viral replication which is correlated to CD4-T cell count and we highlighted that nearly half of those with CD4 count ≥ 500 cells/mm^3^ have a viral replication. For patients receiving ART, we described the viral suppression over time and highlighted the fact that one patient out of five with CD4-T cell count >500 cells /mm^3^ had a detectable viral load, indicating treatment failure. This study confirmed the low viral replication already reported in treatment naïve HIV-2-infected individuals [6,14,28], as less than 10% of them had their viral load >1000 cps/mL and nearly 50% had it <10 cps/mL. This low viremia (31.8 cps/mL in median), associated with the lack of sensitivity of most of the previously described assays is the main reason justifying the non-use of viral load for the monitoring of HIV-2-infected patients on ART [1].

The use of viral load to monitor ART response among HIV-1-infected patients is a common recommendation of WHO already implemented in resources constrained settings [8]. Regarding HIV-2 infection, there was no internationally recognized HIV-2 viral load quantification assay for routine practice and only in-house techniques were used with thresholds ranging between 10 and 1000 cps/mL [6,15,19,29,30]. However, with this ultrasensitive procedure validated in France [26] and allowing thresholds of 10 cps/mL when 1 ml of plasma is available, it may be possible to accurately monitor HIV-2-infected patients receiving ART. In this study, one quarter of patients on ART had a viral load between 10 and 1000 cps/mL, most of them ranged between 10 and 100 cps/mL. Using this ultrasensitive quantification procedure, it may be possible to detect viral replication among HIV-2-infected individuals receiving ART. Usually, the monitoring of HIV-2-infected individuals relies only on CD4-T cell count [4,5,31], and there is no consensus on the definition of immunological failure for the treatment of HIV-2 infection. Given that the immunological failure is most likely due to a suboptimal CD4 count recovery reported in HIV-2-infected patients, there is a concern on using only CD4-T count for the monitoring of these patients [4,5]. Therefore, the use of an ultra-sensitive threshold in association with CD4-T cell count could help in guiding the decision to switch ART regimens. Indeed, by detecting tiny viral load variations, this tool could be of major interest for clinicians, since it allows early detection of drug resistance or treatment non-adherence [25].

The successful ART scaling up [12] and the demonstrated benefits of treatment as prevention in the community [8] have led to the ART guidelines being upgraded. Currently, it is recommended to initiate ART in HIV-infected individuals with CD4 count <500 cells/mm^3^ according to 2013 WHO guidelines [8] or in all patients including those with CD4 count ≥500 cells/mm^3^ according to 2013 French and US guidelines [32]. The aims of this early ART initiation was first to reduce HIV transmission, second to reduce AIDS and non-AIDS related morbidity and mortality [8,32,33] and finally to maintain an undetectable viral load below the threshold of 50 cps/mL among HIV-1-infected patients. It is difficult to duplicate this reduction of HIV transmission in HIV-2 infected individuals since 31% to 40% of these patients already had undetectable (threshold of 100 cps/mL) plasma HIV-2 viral load before ART initiation [28]. The decision of ART initiation among these patients therefore rely only on CD4-T cell count (<500 cells/mL). In our study, 46.7% of ART-naïve individuals with CD4-T cell count ≥ 500 cells/mm^3^ had a detectable viral load (≥10 cps/mL) and among this group of patients, 81.5% had viral load between 10 and 100 cps/mL, making them undetectable with less sensitive assays. Using an ultrasensitive assay could help detect viral replication in these patients who will therefore be eligible for ART according to the latest French guidelines [32].

Despite the clinical importance of these results among HIV-2 infected individuals, our study presents some weaknesses. The genetic diversity of HIV-2 with 9 groups [1,34] was not considered in this study, although it could have an influence on the plasma HIV-2 viral load quantification. However, this genetic diversity of HIV-2 could be weighted by the fact that the large majority of the HIV-2 epidemic in West Africa and overseas is due to two major HIV-2 groups (A and B), which were most prevalent in our population as previously reported [25]. Moreover, the primers and probes used for our HIV-2 RNA quantification assay have been designed to accurately quantify these groups[17]. The results of the quantification of plasma HIV-2 viral load are known to be influenced by the assay and the laboratory procedure as shown in the ACHIEV_2_E collaboration [19]. Thus the availability and the operational use of this assay in resource constrained settings especially West Africa (epicenter of the epidemic) may be challenging. To reduce the variability of the results due to quantification assay and laboratory procedure, the samples of this study had all been tested in the same laboratory following a standardized and validated procedure. The implementation of this new ultrasensitive procedure in West African resource constrained settings is already ongoing. This process includes the training of local staff and frequent quality control with European laboratories. Regarding patients with CD4 >500 cells/mm^3^ and a detectable viral load after treatment, since our study was a cross-sectional survey, we did not perform a CD4-T count control.

The main lesson learned from this study is that the clinical use of viral load is clearly different in treatment-naïve and in ART-receiving patients. In treatment naïve patients, the detection of viral load improved knowledge on the natural history of the infection, showing that more treatment-naïve patients have a viral replication. This finding is important because it could help guiding treatment initiation in these patients. Regarding patients on ART, viral load measurement could help to monitor the treatment efficacy, detect treatment failure and help to reinforce observance. Our results also emphasize the need of an ultrasensitive assay to quantify HIV-2 viral load.

## Conclusion

This study highlights the relationship between plasma HIV-2 viral load and CD4-T cell count in HIV-2-infected individuals. It suggests that the combination of CD4-T cell count and ultrasensitive HIV-2 viral load quantification with a threshold of 10 cps/mL, could improve both ART initiation decision for HIV-2 treatment-naïve patients and the monitoring of ART response among HIV-2-infected individuals.

## Supporting Information

S1 Dataset Dataset including data of the main variables used for the analyses of the study. (XLSX)
